# Characteristics and Injury Patterns in Traumatic Brain Injury Related to E-Scooter Use in Riga, Latvia: Multicenter Case Series

**DOI:** 10.3390/medicina60040540

**Published:** 2024-03-27

**Authors:** Agnis Saulitis, Evelina Kocane, Julija Dolgopolova, Ritvars Kalnins, Kaspars Auslands, Kristaps Rancans, Egils Valeinis, Andrejs Millers

**Affiliations:** 1 Department of Neurology and Neurosurgery, Riga Stradins University, 1007 Riga, Latvia; 044632@rsu.edu.lv (E.K.); kaspars.auslands@rsu.lv (K.A.); andrejs.millers@rsu.lv (A.M.); 2 Department of Neurosurgery, Pauls Stradins Clinical University Hospital, 1002 Riga, Latvia; julija.dolgopolova@stradini.lv (J.D.); kristaps.rancans@stradini.lv (K.R.); egils.valeinis@stradini.lv (E.V.); 3 Department of Neurosurgery, Children’s Clinical University Hospital, 1004 Riga, Latvia; ritvars.kalnins@bkus.lv; 4 Department of Neurosurgery, Riga East Clinical University Hospital, 1038 Riga, Latvia

**Keywords:** electric scooter, e-scooter, traffic accident, craniofacial trauma, traumatic brain injury, neurosurgery

## Abstract

*Background and Objectives:* In recent years, electronic scooters (e-scooters) have gained popularity, whether for private use or as a publicly available transportation method. With the introduction of these vehicles, reports of e-scooter-related accidents have surged, sparking public debate and concern. The aim of this study was to analyze the epidemiological data, characteristics, and severity of traumatic brain injury (TBI) related to e-scooter accidents. *Materials and Methods:* This retrospective case series evaluated patients who were admitted to the three largest neurosurgery clinics in Riga, Latvia, from the time period of April to October in two separate years—2022 and 2023—after e-scooter-related accidents. The data were collected on patient demographics, the time of the accident, alcohol consumption, helmet use, the type of TBI, other related injuries, and the treatment and assessment at discharge. *Results:* A total of 28 patients were admitted with TBI related to e-scooter use, with a median age of 30 years (Q1–Q3, 20.25–37.25), four individuals under the age of 18, and the majority (64%) being male. In 23 cases, the injury mechanism was falling, in 5 cases, collision. None were wearing a helmet at the time of the injury. Alcohol intoxication was evident in over half of the patients (51.5%), with severe intoxication (>1.2 g/L) in 75% of cases among them. Neurological symptoms upon admission were noted in 50% of cases. All patients had intracranial trauma: 50% had brain contusions, 43% traumatic subdural hematoma, and almost 30% epidural hematoma. Craniofacial fractures were evident in 71% of cases, and there were fractures in other parts of body in three patients. Six patients required emergency neurosurgical intervention. Neurological complications were noted in two patients; one patient died. *Conclusions:* e-scooter-related accidents result in a significant number of brain and other associated injuries, with notable frequency linked to alcohol influence and a lack of helmet use. Prevention campaigns to raise the awareness of potential risks and the implementation of more strict regulations should be conducted.

## 1. Introduction

The electric scooter, or e-scooter, has gained popularity as a preferred method of short-distance transportation, whether for public or private use. Its usage has seen a significant uptick in recent years since its initial release in Santa Monica, California, in 2017. Introduced into our social lives under the concept of micro-mobility, the utilization of these vehicles has seen a notable increase during the COVID-19 pandemic [1]. Correspondingly, reports of e-scooter accidents have surged, sparking public debate and concern.

The frequency and severity of accidents associated with electric scooters have become a notable topic, with some medical professionals arguing that the rising usage has led to an escalation in both the occurrence and seriousness of these incidents [1]. Various studies conducted in countries such as Sweden [2], the United States [3,4,5], Germany [6], Austria [7], New Zealand [8], and Turkey [9] have shed light on the impact of e-scooter-related trauma. For instance, research conducted in Sweden has revealed a correlation between e-scooter-related injuries and alcohol consumption, particularly prevalent during the nighttime [2]. In different studies, such as those in Germany, researchers have not only examined the prevalence of head injuries associated with e-scooter accidents but have also explored traumatic injuries affecting other body parts, with a greater frequency noted in the upper limbs [6]. Notably, in Latvia, investigations specifically examining head injuries associated with e-scooters have not been previously undertaken.

Factors such as high speed, low fall height, and short reaction time, coupled with the absence of adequate personal protective equipment, contribute to the heightened vulnerability of e-scooter users, particularly in terms of upper extremity and head injuries. Operating an electric scooter under the influence of alcohol is prohibited in numerous countries, yet there is limited research examining the correlation between alcohol consumption and electric scooter accidents. However, a recent study conducted in New Zealand revealed that up to 27% of accidents were linked to alcohol use [10].

According to the Road Traffic Laws of the Republic of Latvia, individuals aged 14 and above may use e-scooters with a bicycle or another vehicle category license. Consequently, e-scooter sharing service providers are mandated to conduct identity checks on service recipients, including age verification, through their mobile applications or websites. In Latvia, there are no dedicated lanes for e-scooters, so riders share bicycle paths. The maximum permitted speed is 25 km/h, although municipalities reserve the right to set individual speed limits. For instance, in Riga’s parks, the capital of Latvia, the maximum speed is 15 km/h, while in certain areas, such as near educational institutions, it does not exceed 10 km/h. While operating an e-scooter, the driver must maintain a blood alcohol concentration below 0.5 g/L.

Instances of multiple individuals using a single e-scooter have been observed, but legally, e-scooters are designated for single-person use. In Latvia, drivers of e-scooters must wear a protective helmet up to the age of 17, and after that age, it is a person’s free choice whether to wear a helmet or not. The law also explicitly prohibits using a phone or any smart device while operating an e-scooter unless in hands-free mode. Recently, approved amendments to the Vehicle Registration Regulations state that until 31 March 2024, e-scooters can travel without pre-obtained registration stickers. After this date, they must display a state registration sticker to be allowed on the road.

The primary aim of this study was to analyze the epidemiological data, characteristics, and severity of traumatic brain injury (TBI) in patients hospitalized to three neurosurgery clinics in Latvia—Riga East Clinical University Hospital, Riga, Latvia; Pauls Stradins Clinical University Hospital, Riga, Latvia; Children’s Clinical University Hospital, Riga, Latvia.

## 2. Materials and Methods

This three-center retrospective case series study was based on demographic- and health-related data of all patients who were admitted to Departments of Neurosurgery in three university hospitals—Pauls Stradins Clinical University Hospital (PSCUH), Children’s Clinical University Hospital (CCUH), and Riga East Clinical University Hospital (RECUH) after an e-scooter-related accident with recorded intracranial trauma during two selected time periods from 1 April 2022 to 31 October 2022 and 1 April 2023 to 31 October 2023. We selected the time period from the 1st of April to the 31st of October as these were the months when e-scooters were available for public use in Riga and the use of e-scooters on the streets was more common. Since we had to check for the trauma mechanism in individual records of all the patients who had suffered traumatic brain injury in these months, we chose this exact time period as it was more likely to come across patients with e-scooter-related accidents. In PSCUH and RECUH, patients 18 years and older were treated; in CCUH—under the age of 18. All the cases where traumatic brain injury according to ICD-10 S06 was noted, that were admitted to the neurosurgery department, and where the trauma mechanism was related to e-scooter use were included in our study. Patients with injuries not related to electric scooter use and patients without noted traumatic brain injury according to the ICD-10, or incomplete records, were excluded. The patient records of PSCUH, RECUH, CCUH were searched for using the ICD-10 codes for TBI S06 and related subcategories; the cause of accident was identified in individual patient records. A total of 31 patients with TBI related to e-scooter use were admitted to the Departments of Neurosurgery in PSCUH, RECUH and CCUH; 3 patients were excluded, 1 patient did not have TBI, and 2 patient records were incomplete.

All data were extracted from the physical medical records and files stored in the clinical database system “Ārsta birojs” in PSCUH and RECUH, and “Andromeda” and “Saule” in CCUH. The relevant information included sociodemographic data, year, month, day, and time of trauma, mechanism of trauma, Glasgow coma scale (GCS) score upon admission, neurological symptoms upon admission, alcohol intoxication (g/L), whether the patient experienced loss of consciousness or amnesia, helmet status at the time of trauma, seizures after the trauma or during hospitalization, TBI based on ICD-10, craniofacial and other diagnosed fractures, neurosurgical treatment and the type of neurosurgical treatment received during admission, information on whether the patient was stationed in the intensive care unit, received sedation or was intubated, length of hospital stay (days), GCS score at discharge, neurological and general complications upon discharge, disposition, and cause of death.

Descriptive statistics were utilized to determine the frequencies and percentages for dichotomous variables, the mean and median values, standard deviation, and ranges of numerical variables. The distribution of continuous variables was described as the mean and standard deviation for normally distributed variables. Median and interquartile ranges were used to report normally distributed variables. The distribution of the categorical data was reported as numbers and percentages. The data analysis was performed using SPSS 29.0.

## 3. Results

A total of 28 patients were included. A total of 17 patients were admitted in the year 2022 and 11 patients were admitted in 2023. The median age of the patients was 30 years (Q1–Q3, 20.25–37.25) with four individuals being under the age of 18. The gender distribution showed that less than half of the patients were female (35.7%, *n* = 10), while the majority were male (64.3%, *n* = 18). Between April and October, the peak in hospitalizations occurred in August, constituting the highest proportion at 21.4% (*n* = 6), while the remaining months averaged a hospitalization rate of 13.1% for patients. When comparing days of the week, it was observed that only a quarter of the patients (*n* = 7) were hospitalized from Monday to Thursday, while 75% were admitted from Friday to Sunday. Specifically, 25% (*n* = 7) of the patients were hospitalized on Friday, 18% (*n* = 5) on Saturday, and Sunday recorded the highest number of admissions with 32% (*n* = 9). The distribution of hospitalizations remained consistent between day hours (from 7.00 a.m. to 6.59 p.m.) and night hours (from 7 p.m. to 6.59 a.m.) with an equal count of 14 patients in each time period.

In 23 cases, the injury mechanism was a fall, while 5 cases were the result of collisions with vehicles (specifically cars in all instances). None of the patients admitted to the hospital were wearing a head helmet at the time of the injury. Loss of consciousness at the time of injury was observed in 11 patients (39.3%).

Alcohol intoxication was identified in over half of the patients (*n* = 16, 51.1%). Notably, alcohol intoxication was exclusively present in patients aged 18 and above, with no instances observed in the pediatric population. Among the cases with alcohol intoxication, mild levels (<0.5 g/L) were found in 3 patients, moderate levels (0.5–1.2 g/L) in 1 patient, and severe levels (>1.2 g/L) in 12 patients, constituting 75% of all patients who were directly under the influence of alcohol. Overall, 66.7% of all adult patients were diagnosed with alcohol intoxication. The specifics of patient characteristics are shown in Table 1.

Table 2 outlines the neurological and other symptoms observed at admission. When evaluating patients at the emergency department of the hospital, neurological symptoms were observed in 50% of cases. According to the Glasgow coma scale (GCS), 27 patients had mild head injuries with GCS scores ranging from 13 to 15, and among them, 19 patients achieved the maximum GCS score of 15 points. Only one patient had a severe head injury with a GCS score of 3.

Amnesia was the most frequently observed neurological symptom during the pre-hospital or hospital stages, affecting 9 out of 14 patients (32.1%). Other neurological symptoms included vertigo in five patients, cranial nerve palsies in four patients, seizures during the prehospital or hospital stage in three patients, motor aphasia in two patients, sensory aphasia in one patient, paresis in one patient, and nystagmus in one patient. No patients exhibited sensory deficits.

All patients included in the study had traumatic brain injury: 50% had brain contusion, 42.9% had traumatic subdural hematoma, 28.6% had epidural hematoma, 17.8% had traumatic subarachnoid hematoma, and 17.8% had concussion. Craniofacial fractures were found in 20 patients (71%), who had combined fractures in different regions of the skull—50% had cranial vault fractures, 46.4% had skull base fractures, and 46.4% had facial bone fractures.

Fractures impacting other body regions were observed in three patients, involving upper extremity fractures (7.1%), rib fractures (7.1%), and vertebral fractures (7.1%). No lower extremity fractures were detected in any patient. Vertebral fractures of the spine were identified in two patients (7.1%), with one experiencing a cervical spine fracture and the other having a lumbar spine fracture. Additionally, soft tissue lesions like skin abrasions were prevalent in the majority of patients (*n* = 18, 64.3%). Table 3 provides a summary of all the data pertaining to injuries.

Over one-fifth of the patients (*n* = 6, 21.4%) required specific neurosurgical intervention; craniotomy was performed to evacuate the hematoma, but one of these patients also underwent a secondary decompressive craniectomy. Among them, two patients were admitted to the intensive care unit and received sedation, while the patient undergoing decompressive craniectomy was the only one requiring intubation outside of the operative setting. A detailed review of each patient individually is presented in Table 4.

The hospitalization duration ranged from a minimum of 2 days to a maximum of 45 days, with a median length of stay at 5.5 days. Among the discharged patients (*n* = 27) for further treatment, 25 patients had a GCS score of 15 points, and 2 patients had a GCS score of 14 points. A comparison of GCS scores at admission and discharge revealed that six patients initially scored below 15 points but achieved the maximum GCS score of 15 points upon discharge. Additionally, one patient consistently scored 14 points at both admission and discharge, and one patient demonstrated improvement from a GCS score of 13 at admission to 14 at the time of discharge.

Neurological complications were noted in two patients, both during their inpatient stay and at the time of discharge. One patient experienced cranial nerve palsies and two patients exhibited motor aphasia. Notably, these were the only individuals with a GCS score of 14 at the time of discharge. No general complications were noted upon discharge.

Following the outcome, 26 patients were released to their homes, 1 patient was moved to a lower-level hospital for additional care, and 1 patient passed away.

## 4. Discussion

The rising popularity of e-scooters has been accompanied by a growing number of e-scooter-related injuries. This study represents the first examination of e-scooter accidents in Latvia. Our objective was to analyze the epidemiological data, as well as the characteristics and severity of traumatic brain injuries in patients admitted to three neurosurgery clinics in Latvia. Additionally, findings were compared with existing data from other countries.

In numerous studies, it has been consistently highlighted that injuries related to e-scooter usage predominantly affect men [2,3,4,6,7,9,11,12,13,14]. Our findings align with this trend, revealing that 64.3% of hospitalized patients were men, whereas only 35.7% were women. This pattern is also reflected in cases where patients were under the influence of alcohol at the time of injury—13 of the patients were men and only 3 were women. This suggests a higher likelihood of men using e-scooters while intoxicated, potentially contributing to the notable male predominance observed. It is worth noting that Blomberg et al. (2019) [15] stand out, reporting in their study that 57.1% of all e-scooter-related injury patients were female. Similar findings were reported by Büyükceran et al. (2023) [16], where their study revealed that 50.5% of patients were female, while 49.5% were male.

Taking into account the time of day, other studies by various authors have noted an increased occurrence of e-scooter accidents during evening and nighttime hours, contrary to our findings [7,12,15]. Moftakhar et al. (2021) [7] observed that 58.3% of all e-scooter-related admissions took place between 8:00 p.m. and 7:59 a.m. Trivedi et al. (2019) [12] reported that 75% of accidents happened between 3 p.m. and 7 a.m. These findings did not align with the outcomes of our study, where half of the patients were hospitalized during the day (7:00 a.m. to 6:59 p.m.) and the other half during the night (7:00 p.m. to 6:59 a.m.).

In this study, there were only four pediatric patients (under 18 years of age) with a mean age of 13.8 years (range, 12–15 years). Several studies have specifically delved into the pediatric population concerning e-scooter injuries [13,14,16]. For instance, Cohen et al. (2022) [13] noted that children exhibit a higher incidence of fractures and polytrauma related to e-scooters when compared to adults, although they experience fewer facial injuries despite a similar rate of head trauma. In another study, Morgan et al. (2022) [14] reported findings on 10 patients, among whom 5 necessitated orthopedic surgery. However, Büyükceran et al. (2023) [16] incorporated 49.4% of patients below 18 years and 50.6% of patients aged 18 and above in their research. Their findings indicated that pediatric patients were more prone to upper extremity injuries, whereas adults were more predisposed to lower extremity injuries.

It is crucial to highlight the importance of employing protective gear while riding e-scooters. Numerous studies have attested to the limited use of helmets in the context of e-scooter usage [2,4,5,6,9,11,12,13,14,15,17,18]. This observation is consistent with the data obtained in our study, where none of the participants, including pediatric patients, were wearing helmets at the time of injury. In our study, a direct comparison of outcomes between patients wearing and not wearing helmets was not possible. However, there are studies that have assessed the effectiveness of helmets through simulations with human body models. The results of these studies suggested that protective helmets can mitigate the force of impact during injuries, thus reducing the risk of head injuries [19,20,21]. It is worth noting that as of 1 January 2024, amendments to the Road Traffic Regulations of the Republic of Latvia mandate that all e-scooter drivers under the age of 17 must wear a protective helmet. However, for individuals beyond this age, the use of a protective helmet is only recommended.

One fatal outcome was recorded in our case series. The patient was a 30-year-old male; although it has not been proven that men have a higher risk of fatal outcomes in this scenario, there have been studies showing male predominance in fatal outcomes related to e-scooter use [22]. Previous data also showed role in factors such as time of the day—with more fatal cases occurring during nighttime (from 10 p.m. to 5 a.m.), and regarding alcohol intoxication, in our case, the trauma occurred at around 5 a.m., since the patient arrived at the hospital at 05.46 a.m., and the patient was under the influence of alcohol, with severe intoxication evident upon arrival at the emergency department (1.2 g/L) [22]. Upon admission, the patient’s GCS score was 3, although it is not possible to generalize this data—it could be a factor for worse prognosis, as the patient’s overall condition was initially poor. Also, in this case, the patient needed a second neurosurgical intervention—a decompressive craniectomy after the initial craniotomy and an epidural hemorrhage evacuation, which shows that the damage to the brain was extensive.

Our study revealed one patient with a fatal outcome. However, an analysis of clinical cases from other authors highlighted similar tragic incidents. One case was described by Aulino et al. (2022), in which a 33-year-old man was involved in a fatal e-scooter accident [23]. Following a frontal collision with a car while driving a shared e-scooter, the man sustained severe cranioencephalic injuries. Despite medical attention, he succumbed to his injuries half an hour after the incident. The autopsy revealed multiple fractures of the skull vault and skull base, along with intracranial bleeding including subdural hematoma above the left-brain hemisphere, a subarachnoid hemorrhage in the bilateral parietal lobes and cerebellar hemispheres, as well as an intraventricular hemorrhage. In addition, lacerations focused in the cerebellum and brainstem were also discovered. In another study by Karpinski et al. (2022), which examined 21 deaths in the US attributable to e-scooter use from 2018 to 2020, it was concluded that over 80% of cases resulted from collisions with motorized vehicles, often occurring during nighttime or adverse weather conditions such as precipitation or fog. The most common accident scenarios involved motor vehicles colliding with e-scooters from behind or e-scooter riders losing control. In the majority of instances, victims sustained severe head or brain injuries [24].

When considering deaths due to cranioencephalic trauma, including those involving electric scooter accidents, it is important to analyze structural deficiencies and compare them with other cases to understand commonalities and potential preventive measures. Structural deficiencies can include various factors such as inadequate infrastructure, lack of safety regulations, and behavioral patterns contributing to accidents such as the aforementioned alcohol consumption while driving an e-scooter.

In order to reduce injuries and possible deaths caused by the misuse of e-scooters, the public should be encouraged to use a safety helmet. Firstly, there should be educational campaigns telling people about the severity of head injuries and deaths that have occurred in accidents where safety helmets were not used. Wei W. et al. (2022) pointed out that helmet use is effective in reducing head injury rates but not preventing severe head injuries [19]. However, in our study, we concluded that head injuries were mostly mild and could be treated conservatively without neurosurgical treatment in most cases. In this way, people should be encouraged to use a helmet and be told about the advantages of using a helmet for the prevention of possible head injuries.

Secondly, it would be valuable to implement measures that legally determine the use of a protective helmet for e-scooter riders. Governments and local authorities can pass rules and laws to make the use of helmets mandatory, as is the case for motorcyclists, for example. Fines for not wearing a safety helmet could encourage compliance with helmet wearing requirements. However, to be able to require e-scooter riders to use helmets during the ride, it would be necessary to offer a convenient way of where and how to obtain those helmets. If a person rides his/her personal e-scooter, then buying an additional protective helmet would not be a problem, but if a person uses shared e-scooters, then there might be difficulties with hygiene if one helmet is used by several people a day. Therefore, it would be valuable to promote cooperation between the e-scooter sharing company and helmet manufacturers, as well as to implement several e-scooter rental stations within the city where helmets could also be rented.

Ultimately, the risk of serious cranial trauma and death can be minimized by wearing a helmet, but implementing them requires a comprehensive strategy that includes education, regulation, accessibility, technology, and cultural change because nothing will change until people change their views.

Our study had certain limitations worth noting. This was a retrospective study, and patients admitted to the neurosurgery department were exclusively included, overlooking individuals who received outpatient treatment for e-scooter-related head injuries. The inclusion of outpatients would allow for comparisons across different severities of head injuries and an exploration into the correlation with helmet usage. Furthermore, a prospective study with an extended data collection period could encompass a larger patient population for a more comprehensive analysis, as this work should be used with caution while generalizing the data.

In this study, we gathered the latest information on head injuries associated with e-scooters in Latvia. The data were collected from the three major neurosurgery centers in the country, examining various factors pertinent to the subject. The findings obtained could potentially contribute to future modifications in legal aspects or the enhanced monitoring of adherence to existing regulations. These include parameters like the allowable alcohol intoxication limit while operating an e-scooter and the requirement for a protective helmet. There is also a requirement to prompt e-scooter sharing companies to meticulously verify customer identity and age, as evidenced by our study, which involved two cases where patients were under the age of 14 using an e-scooter.

## 5. Conclusions

Electric scooters have become a prevalent means of transportation in many urban areas. Accidents involving e-scooters lead to a substantial number of brain and other associated injuries, with notable frequency linked to alcohol influence and a lack of helmet use. It is imperative to conduct prevention campaigns to enhance awareness of potential risks and advocate for the implementation of stricter regulations.

## Figures and Tables

**Table 1:** Patient and traffic accident characteristics.

	*n* (%)
Age in years (mean)	30
Under 18 years of age	4 (14.3%)
Over 18 years of age	24 (85.7%)
Sex	
Male	18 (64.3%)
Female	10 (35.7%)
Mechanism of injury	
Fall	23 (82.2%)
Collision with car	5 (17.8%)
Time of injury	
Daytime (7.00 a.m. to 6.59 p.m.)	14 (50.0%)
Nighttime (7.00 p.m. to 6.59 a.m.)	14 (50.0%)
Helmet use	
No helmet	28 (100.0%)
Wearing a helmet	0 (0.0%)
Alcohol intoxication	
No intoxication	12 (42.9%)
<0.5 g/L	3 (10.7%)
0.5–1.2 g/L	1 (3.6%)
>1.2 g/L	12 (42.9%)

**Table 2:** Neurological symptoms at the emergency department and other symptoms.

	*n* (%)
GCS for traumatic brain injury	
Mild (13–15)	27 (96.4%)
Moderate (9–12)	0 (0.0%)
Severe (3–8)	1 (3.6%)
Neurological symptoms	14 (50.0%)
Cranial nerve palsies	4 (14.3%)
Motor aphasia	2 (7.14%)
Sensor aphasia	1 (3.6%)
Paresis	1 (3.6%)
Sensor deficits	0 (0.0%)
Seizure (pre-hospital or hospital stage)	3 (10.7%)
Amnesia (pre-hospital or hospital stage)	9 (32.1%)
Vertigo	5 (17.9%)
Nystagmus	1 (3.6%)
Other symptoms	
Loss of consciousness at the moment of injury	11 (39.3%)

**Table 3:** E-scooter-related injuries.

	*n* (%)
Traumatic brain injury (patients)	28 (100.0%)
Concussion	5 (17.8%)
Contusion	14 (50.0%)
Epidural hematoma	8 (28.6%)
Traumatic subdural hematoma	12 (42.9%)
Traumatic subarachnoidal hematoma	5 (17.8%)
Craniofacial factures (patients)	20 (71.0%)
Cranial vault	14 (50.0%)
Base of the skull	13 (46.4%)
Facial bones	13 (46.4%)
Fractures (non-head) (patients)	3 (10.7%)
Upper extremities	2 (7.1%)
Lower extremities	0 (0.0%)
Ribs	2 (7.1%)
Vertebrae of the spine	2 (7.1%)
Vertebral fractures (patients)	2 (7.1%)
Cervical	1 (3.6%)
Thoracic	0 (0.0%)
Lumbar	1 (3.6%)
Sacral	0 (0.0%)
Soft tissue injuries (abrasions, skin lesions) (patients)	18 (64.3%)

**Table 4:** Data of patients.

*n*	Sex	Age	Time of the Day	Month	Mechanism of Injury	Alcohol	Alcohol Level in Blood (g/L)	Outcome Treatment
1	m	30	n	August	fall	yes	>1.2	conservative
2	m	48	n	July	fall	no	-	conservative
3	m	38	n	October	fall	yes	>1.2	conservative
4	m	48	d	July	fall	yes	>1.2	conservative
5	m	35	n	August	fall	yes	>1.2	conservative
6	f	28	d	October	fall	no	-	conservative
7	m	41	n	October	fall	yes	>1.2	conservative
8	f	29	n	September	fall	yes	>1.2	conservative
9	f	29	d	August	fall	no	-	conservative
10	m	51	n	April	fall	yes	>1.2	conservative
11	f	19	n	July	fall	yes	>1.2	conservative
12	f	15	d	April	collision	no	-	conservative
13	f	15	d	October	fall	no	-	craniotomy
14	f	13	d	September	collision	no	-	conservative
15	m	12	n	August	fall	no	-	craniotomy
16	m	35	n	September	fall	yes	>1.2	conservative
17	f	35	d	June	fall	no	-	conservative
18	m	31	n	April	fall	yes	>1.2	craniotomy
19	m	30	n	May	fall	yes	>1.2	(1) craniotomy, (2) decompressive craniectomy. Fatal outcome
20	m	64	n	August	fall	yes	<0.5	craniotomy
21	m	38	d	September	collision	no	-	conservative
22	m	35	d	August	fall	yes	<0.5	conservative
23	m	31	d	May	fall	no	-	conservative
24	m	30	d	May	fall	yes	0.5–1.2	craniotomy
25	f	21	n	April	fall	yes	>1.2	conservative
26	m	18	n	June	collision	no	-	conservative
27	m	20	d	May	fall	yes	<0.5	conservative
28	f	24	n	June	collision	no	-	conservative

Abbreviations: m—male, f—female, d—daytime (7.00 a.m. to 6.59 p.m.), n—nighttime (7.00 p.m. to 6.59 a.m.).

## Data Availability

Data are available upon request due to ethical restrictions. All the data included in this study are available upon request from the corresponding author. The data are not publicly available and are stored in the patient medical record repository at Pauls Stradiņš Clinical University Hospital, Children’s Clinical University Hospital, and Riga East Clinical University Hospital according to where the individual patient was treated.
